# Supplementary material for: Discovery of Small-Molecule Activators for Glucose-6-Phosphate Dehydrogenase (G6PD) Using Machine Learning Approaches
Source: Int J Mol Sci. 2020 Feb 23;21(4):1523. doi: 10.3390/ijms21041523 (PMC7073180; doi:10.3390/ijms21041523)
Supplement: Supplementary file 1 [file ijms-21-01523-s001.zip › Supplementary_Data.docx]

Article

Discovery of small molecule activators for G6PD (glucose-6-phosphate dehydrogenase) using machine learning approaches (Supplementary data)

Madhu Sudhana Saddala, Anton Lennikov and Hu Huang*

Mason Eye Institute, University of Missouri School of Medicine, Columbia, Missouri, USA.

***** Correspondence: huangh1@missouri.edu; Tel.: +1-573-882-9899 (H.H.)

**Supplementary Figure 1:** CASTp active site prediction of G6PD molecule and it’s features.


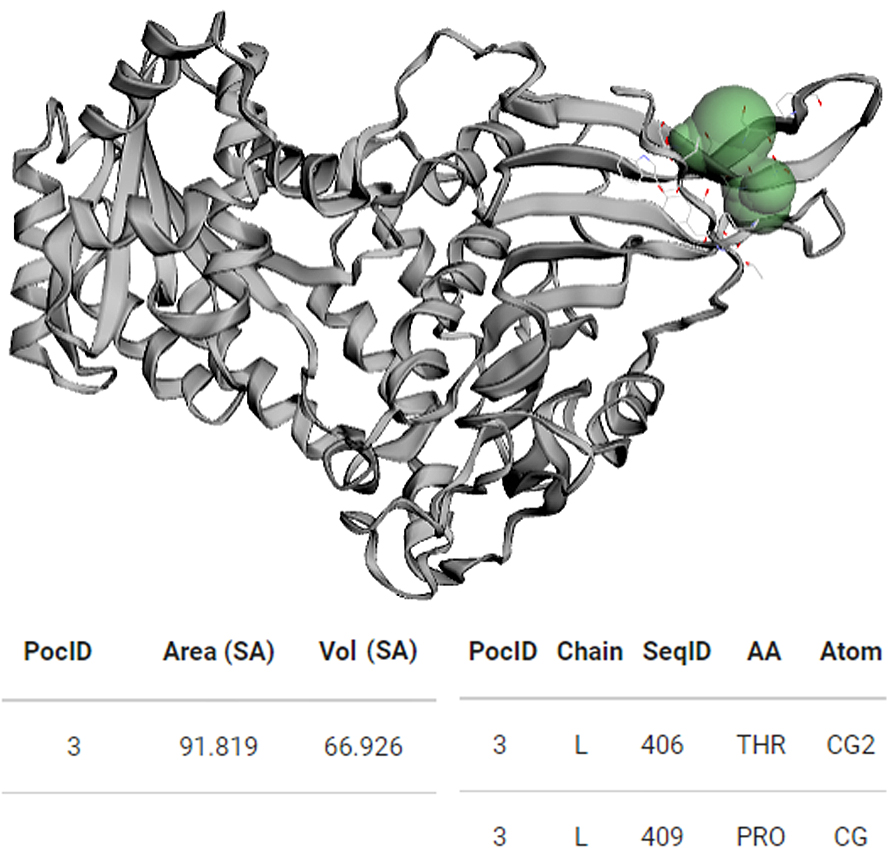


**Supplementary Figure 2:** Molecular structure of 53 compounds candidates selected for machine learning analysis. A raw chemical table file is attached as a supplementary file 04_53_hits.sdf


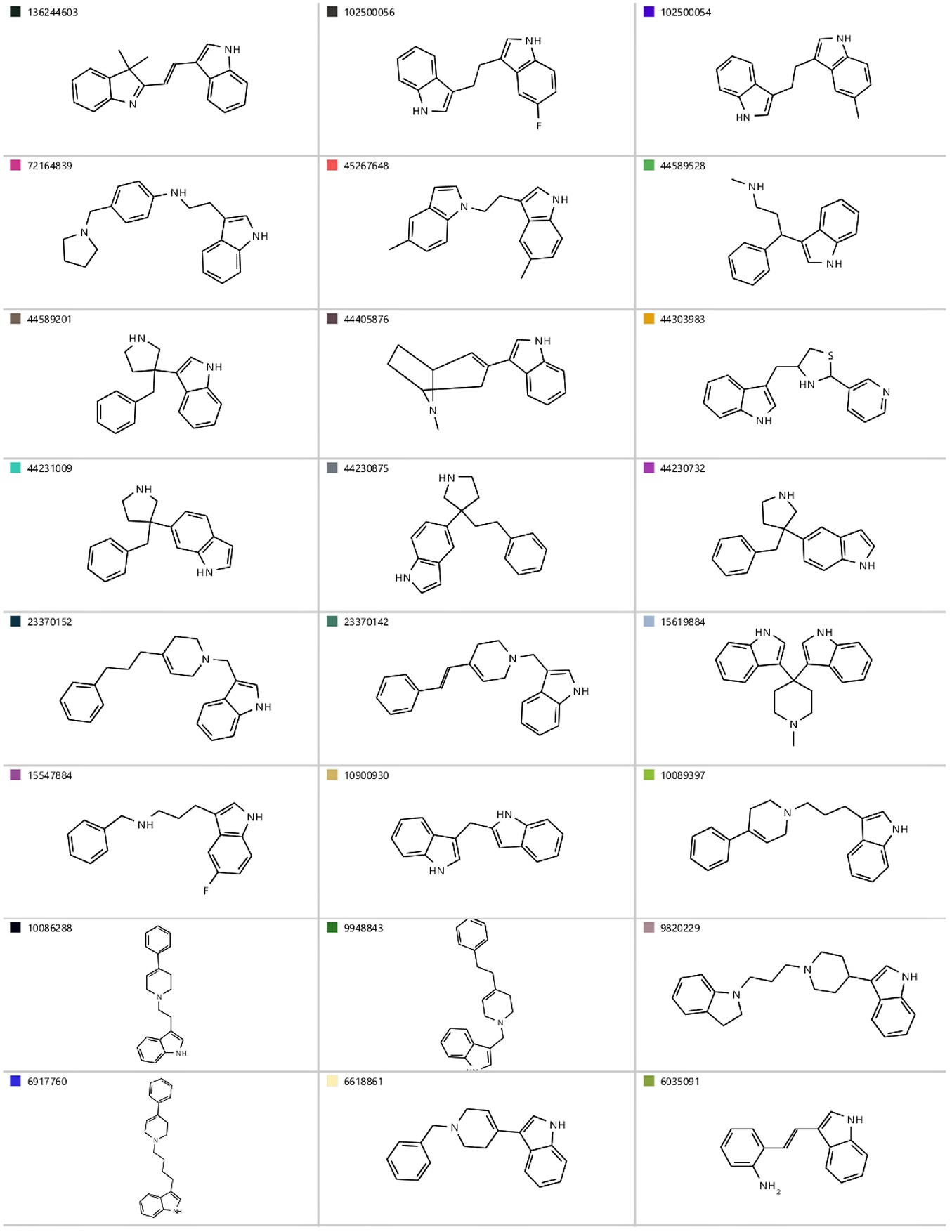


**
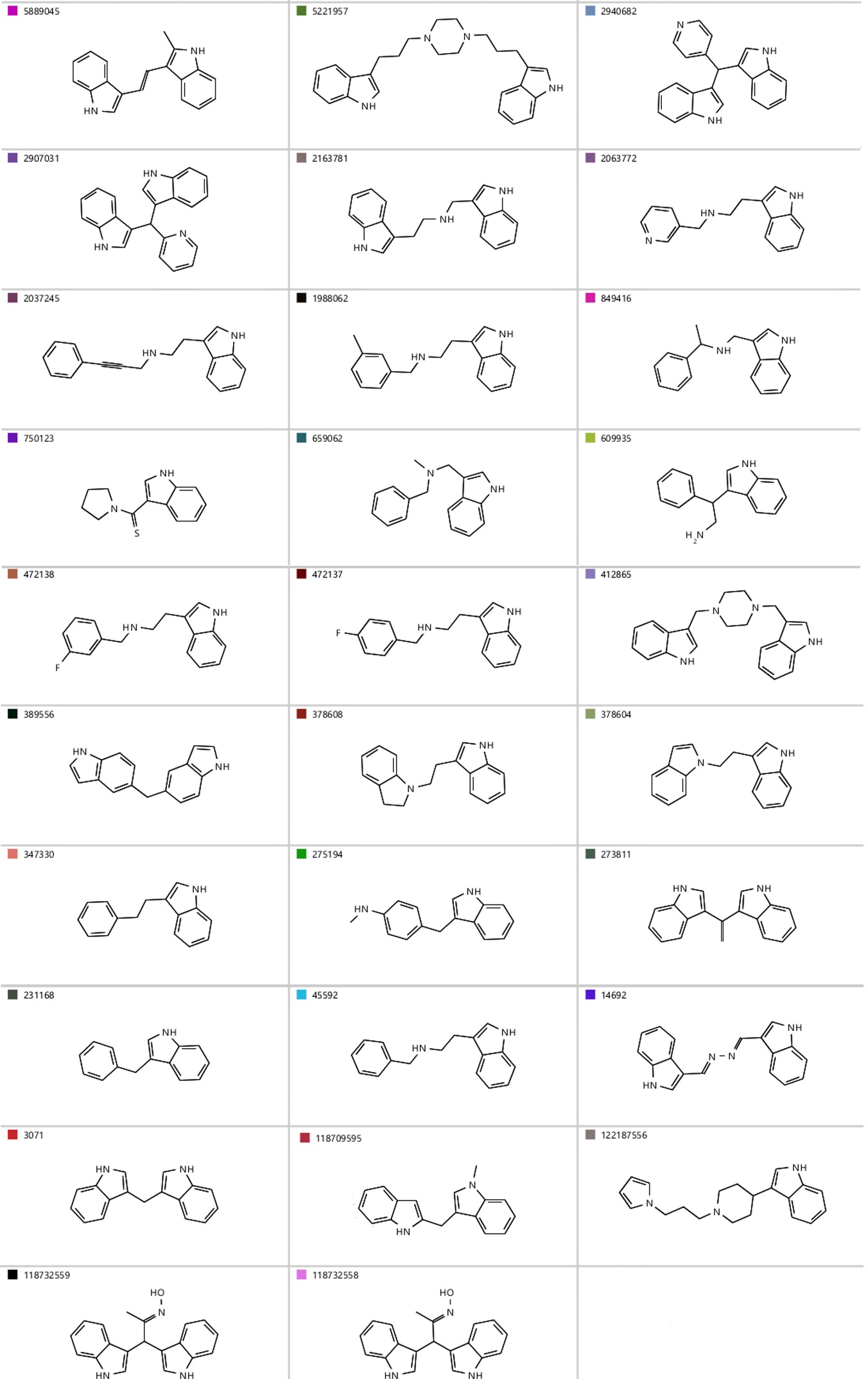
**

| 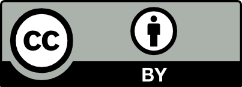 | © 2019 by the authors. Submitted for possible open access publication under the terms and conditions of the Creative Commons Attribution (CC BY) license (http://creativecommons.org/licenses/by/4.0/). |
| --- | --- |
